# Automated manufacturing of clinical-grade BDCA2 CAR NK cells in a closed system for the treatment of blastic plasmacytoid dendritic cell neoplasm

**DOI:** 10.3389/fimmu.2026.1761397

**Published:** 2026-02-27

**Authors:** Rita Pfeifer, Sabine Müller, Marcus Nitsche, Melanie Sohmen, Julia Kostyra, Arthur Bister, Cathrin Bleilevens, Janina Brauner, Angela Mekes, Juliane Raasch, Dominika Lukas, Jens Kopatz, Wael Al Rawashdeh, Marsilius Mues, José Alberto Villacorta Hidalgo, Volker Huppert, Mario Assenmacher, Nina Möker, Rimas Orentas, Congcong Zhang

**Affiliations:** 1 Miltenyi Biotec B.V. & Co. KG, Bergisch Gladbach, Germany; 2 Lentigen Technology, Inc., a Miltenyi Biotec Company, Gaithersburg, MD, United States

**Keywords:** automated manufacturing, BDCA2 CAR, BPDCN, cancer immunotherapy, CAR NK cells, CliniMACS Prodigy NKCT

## Abstract

Recent progress in chimeric antigen receptor (CAR) natural killer (NK) cell therapy has demonstrated their promising potential in cancer immunotherapy. However, most current CAR NK cell manufacturing processes utilize open systems with multiple manual steps, making it challenging to maintain consistent therapeutic quality and regulatory compliance for clinical applications. We specifically developed blood dendritic cell antigen 2 (BDCA2)-targeting CAR NK cells for treating blastic plasmacytoid dendritic cell neoplasm (BPDCN). Here, we present an automated, current good manufacturing practice (cGMP)-compliant Natural Killer Cell Transduction (NKCT) process for producing clinical-grade CAR NK cells on the CliniMACS Prodigy^®^ platform. This closed system integrates cell separation, activation, transduction, expansion, and harvest, thereby reducing contamination risks and ensuring cell product quality. The NKCT process achieved high transduction efficiency using baboon envelope pseudotyped lentiviral vectors (BaEV-LV) produced under cGMP conditions combined with Vectofusin^®^-1, yielding CAR NK cells with high viability and purity. Both *in vitro* and *in vivo* studies demonstrated the potent antitumor activity of CliniMACS Prodigy-manufactured BDCA2 CAR NK cells, highlighting a promising treatment strategy for BPDCN. In summary, this automated NKCT process enables both centralized and decentralized CAR NK manufacturing and facilitates the efficient clinical translation of CAR NK cell therapies.

## Introduction

Natural killer (NK) cells have the inherent ability to recognize and eliminate virus-infected cells and malignant cells without the need for prior sensitization (1, 2). The safety and efficacy of harnessing NK cells to treat cancers in the allogeneic setting has been demonstrated in clinical trials (3). In recent years, remarkable advances have been made in using genetically engineered NK cells, especially chimeric antigen receptor (CAR)-engineered NK cells, in cancer immunotherapy (4). The first-in-human clinical trial of CD19 CAR NK cells has shown promising results against B cell malignancies (5). The authors further demonstrated that favorable clinical outcomes correlated with the persistence of CAR NK cells and defined the selection criteria of allogeneic donors for CAR NK cell production (6).

Manufacturing CAR NK cells is a complex process and ensuring consistent high quality of the cell product for clinical applications. Most current CAR NK cell manufacturing processes are largely based on open systems with multiple labor-intensive, manual steps that are prone to operator-introduced errors and product contamination. Translating such processes into current good manufacturing practice (cGMP)-compliant ones that meet regulatory requirements and enable a second site to manufacture the same CAR NK cell products, thus enabling true point-of-care manufacturing, can be challenging. An automated manufacturing process in a closed system can markedly reduce operator hands-on time and ensure consistency and high quality of the final CAR NK cell products. Previous automated processes for NK cell separation and expansion using the CliniMACS Prodigy platform still required the genetic modification of NK cells to be performed manually outside of Prodigy devices (7–9). Moreover, the vesicular stomatitis virus G (VSV-G) pseudotyped lentiviral vectors (LV) used in most T cell transduction procedures performs poorly with NK cells. It has demonstrated that high transduction efficiency of NK cells can be obtained using baboon envelope pseudotyped lentiviral vectors (BaEV-LV) (10, 11). BaEV-LV-based transduction of NK cells can be further enhanced by the addition of an amphipathic transduction-enhancing agent, Vectofusin-1 (10). The simplified NK cell transduction protocol described herein facilitates the automation of CAR NK cell manufacturing.

Automation of immunotherapy cell product manufacturing has been a driving factor in process development for clinical applications. The automated Natural Killer Cell Transduction (NKCT) process enables the generation of CAR NK cells under cGMP-compliant conditions. The NKCT process covers the entire manufacturing workflow for producing clinical-grade CAR NK cells, starting from cell separation and activation to transduction, expansion and harvest. Blood dendritic cell antigen 2 (BDCA2, CD303, or CLEC4C) is a type II C-type lectin receptor that is highly and selectively expressed on plasmacytoid dendritic cells (pDCs), making it a promising target for treating blastic plasmacytoid dendritic cell neoplasm (BPDCN) (12, 13). Targeting BDCA2 and its associated signaling pathways with monoclonal antibodies and small molecules has yielded encouraging results in preclinical BPDCN studies (14, 15). However, no BDCA2-specific CAR therapy has been developed until now. Here, we present the first development of CAR NK cells targeting BDCA2 for BPDCN treatment. BDCA2 CAR NK cells produced by using the NKCT process on the CliniMACS Prodigy platform demonstrated potent anti-tumor activity *in vitro* and *in vivo*.

## Materials and methods

### Small-scale manual NK cell transduction

Human primary NK cells were isolated from the whole blood of healthy volunteers with written informed consent as approved by the local ethics committee of Ärztekammer Nordrhein (2020272). NK cell isolation was performed using CD3 MicroBeads followed by CD56 MicroBeads according to the manufacturer’s instructions. Freshly isolated human NK cells were primed at a density of 1 × 10^6^ cells/mL in a 24-well plate using NK MACS Medium supplemented with 5% AB serum (Access Biologicals, Vista, CA, USA), 140 U/mL recombinant human IL-15, 500 U/mL recombinant human IL-2, and 2000 U/mL recombinant human IL-1β and cultured at 37°C in a 5% CO2 incubator. Two days after seeding, BaEV-LV, premixed with Vectofusin-1, were added dropwise to the activated NK cells, and gently mixed by pipetting. Spinoculation was then applied at 32°C and 400 × g for 2 hours (h). Vectofusin-1 was used at a final concentration of 2.5 µg/mL. One day after transduction, NK cells were washed to remove excess BaEV-LV and cultured further in NK MACS Medium supplemented with 5% AB serum, 140 U/mL IL-15, and 500 U/mL IL-2. Regular feeding and medium changes were performed every 2–3 days to maintain the cell density below 2 x 10^6^ cells/mL until harvest on day 14. The effects of IL-1β, serum, spinoculation, and Vectofusin-1 was assessed in this study to establish the optimized transduction protocol outlined here.

### Automated generation of genetically modified NK cells

For automated generation of genetically modified NK cells, the NK Cell Transduction (NKCT) process was used on the closed and cGMP-compliant CliniMACS Prodigy platform. NK cells were isolated from leukapheresis, acquired from healthy volunteers, with written informed consent, as approved by the local ethics committees of Hannover Medical School (4718NM), University Hospital Ulm (172/99), and University Hospital Cologne (03-055). NK cell separation was performed by automated immunomagnetic selection. First, T cells were depleted with CliniMACS CD3 Reagent, followed by enrichment of NK cells using CliniMACS CD56 Reagent. For cytokine-based activation of NK cells, 1 x 10^8^ NK cells were seeded in 70 mL of NK MACS GMP Medium supplemented with 5% AB Serum, 140 U/mL MACS GMP recombinant human IL-15, 500 U/mL MACS GMP recombinant human IL-2, and additional 5 mL of MACS GMP recombinant human IL-1β to achieve a final working concentration of 2000 U/mL. Cells were cultivated at 37°C and 5% CO_2_. The pH value was maintained between 7 and 7.8. The temperature was automatically maintained by Prodigy devices. CO_2_ concentration was set by the gassing input controlled by Prodigy devices. Two days after isolation, NK cells were transduced with BaEV-LV encoding BDCA2 CAR (described below). BaEV-LV, premixed with MACS GMP Vectofusin-1, was added to the activated NK cells at a multiplicity of infection (MOI) of 0.4. The final concentration of Vectofusin-1 was 2.5 µg/mL. The NK cells were then subjected to spinoculation at 32°C and 400 x g for 2 h. One day post-transduction, NK cells were washed to remove excess BaEV-LV and subsequently cultured in NK MACS GMP medium supplemented with 5% AB serum, 140 U/mL IL-15 and 500 U/mL IL-2 with automated feeding and medium exchange until harvest on day 14. During the automated cell harvest, NK cells were washed and resuspended in 100 mL of CliniMACS Formulation Solution for subsequent use.

### CAR construction and lentiviral vector production

A second-generation BDCA2-specific CAR was generated using a previously described monoclonal antibody against BDCA2 (13, 16). Briefly, the BDCA2-specific scFv was fused in frame with the CD8 hinge and transmembrane domain, the 4-1BB (CD137) costimulatory domain, and the CD3ζ (CD247) activation domain. A leader peptide derived from GM-CSFRα was included to facilitate CAR cell surface expression. Third-generation self-inactivating BaEV-LV encoding the BDCA2 CAR were produced via transient transfection of HEK293T cells, as described previously (10).

### Flow cytometric analysis of CAR NK cells

Cellular composition and NK cell phenotype were determined by using the following antibodies: anti-human CD45-VioBlue (REA747), anti-human CD14-VioGreen (REA599), anti-human TCRab-FITC (REA652), anti-human CD3-PE (SK7, BioLegend), anti-human CD56-APC (REA196), and anti-human CD19-APC-Vio770 (REA675). BDCA2 CAR expression was analyzed using a biotinylated anti-BDCA2 CAR idiotype antibody, followed by detection with an anti-biotin-Vio Bright B515 antibody (REA746). All staining procedures were performed in the presence of FcR Blocking Reagent. For the exclusion of dead and apoptotic cells, 7-amino-actinomycin D (7-AAD) was used. Data were acquired by using a MACSQuant^®^ Analyzer 10 and analyzed either manually or automatically with NK Cell Express Mode in the MACSQuantify 2.13 software (Miltenyi Biotec, Bergisch Gladbach, Germany).

### Determination of vector copy number

After 14 days of culture, genomic DNA was purified from 1 × 10^6^ harvested CAR NK cells using the DNeasy Tissue Kit (Qiagen, Hilden, Germany). LV encoded *gag* gene copies per genome were determined in relation to the human reference gene *PTBP2* by quantitative PCR using the MACS COPYcheck Kit.

### *In vitro* functional characterization of BDCA2 CAR NK cells

For cytotoxicity analysis, RS4;11 cells engineered to express enhanced green fluorescent protein (EGFP) and firefly luciferase (ffluc) (hereafter RS4;11), or RS4;11 cells additionally expressing human BDCA2 (hereafter RS4;11/BDCA2), were seeded at a density of 2 × 10^4^ cells per well in flat-bottom 96-well plates. Subsequently, either untransduced (UTD) or BDCA2 CAR NK cells were added at the designated effector-to-target (E:T) ratios. Samples containing tumor cells without NK cells served as controls. The long-term *in vitro* antitumor activity of BDCA2 CAR NK cells was assessed using a rechallenge assay. CAR NK cells were co-incubated with 3 x 10^4^ RS4;11/BDCA2 tumor cells at an E:T ratio of 1:2, followed by the addition of 3 x 10^4^ fresh tumor cells every 24 h. Samples were stained with 7-AAD to exclude dead cells after co-incubation and viable GFP-positive RS4;11 cells were quantified using a MACSQuant Analyzer 10. Cytotoxicity of NK cells was determined as (1 - (number of viable GFP-positive cells/number of control GFP-positive cells)) × 100%. To quantify cytokine secretion, tumor cells were co-incubated with either UTD NK or CAR NK cells at an E:T ratio of 1:1 in a flat-bottom 96-well plate for 24 h. Supernatants were then harvested and analyzed using a MACSPlex Cytokine 12 Kit according to the manufacturer’s instructions.

### *In vivo* functional analysis of BDCA2 CAR NK cells

The *in vivo* study was approved by the state animal research committee (LANUV, North Rhine-Westphalia, Germany) and animals were cared for according to guidelines of the Federation of European Laboratory Animal Science Associations. Eight- to ten-week-old female NOD-SCID IL2R γ^null^ (NSG) mice (Charles River Laboratories, Sulzfeld, Germany) were intravenously (i.v.) injected with 5 × 10^5^ RS4;11/BDCA2 cells through the tail vein. Seven days later, mice received an i.v. injection of 1 × 10^7^ CliniMACS Prodigy-manufactured BDCA2 CAR NK cells or an equal number of UTD NK cells. Two groups of animals, treated with either UTD NK cells or BDCA2 CAR NK cells, received daily subcutaneous injections of 25,000 IU IL-2 (Proleukin S; Novartis Pharma, Nürnberg, Germany), starting one day before (day -1) and continuing through nine days after NK cell treatment (day 8). A group of mice that did not receive any NK cells following tumor inoculation served as controls. Tumor load was assessed every 2–3 days by bioluminescence imaging in an IVIS Lumina II system (PerkinElmer, Rodgau, Germany). Mice were anesthetized using 1.5-2% isoflurane in oxygen as the carrier gas during imaging. Luminescence was analyzed using the Living Image software (PerkinElmer, Rodgau, Germany). Upon reaching endpoint criteria or the conclusion of the experiment, mice were euthanized using a CO_2_ gas flow regulator with a control valve appropriate for the cage size (6 L/min for EU Type II long cages and 13 L/min for EU Type III cages). The persistence of tumor cells, NK cells and CAR NK cells in the bone marrow, blood, and spleen was determined by flow cytometry. The following antibodies were used: anti-human CD45-VioBlue (REA747), anti-human BDCA2-PE (AC144), anti-human CD56-APC (REA196), anti-mouse CD45-APC-Vio770 (REA737), anti-mouse Ter119-PE-Vio 770 (REA847), biotinylated anti-BDCA2 CAR idiotype antibody, and anti-biotin-Vio Bright B515 antibody (REA746). Dead cells were excluded by Propidium Iodide (PI).

### Statistical analysis

All statistical analyses were performed using GraphPad Prism 10 software (GraphPad Software, Boston, MA, USA). Data was analyzed using two-tailed paired or unpaired Student’s t tests, or two-way analysis of variance (ANOVA). A *p* value < 0.05 was considered statistically significant.

### Materials and equipment sources

All reagents, tubing set systems, and devices, software used in this study were obtained from Miltenyi Biotec, Bergisch Gladbach, Germany, unless otherwise specified.

## Results

### Automation of NK cell isolation, activation, transduction, and expansion

**Figure 1:**
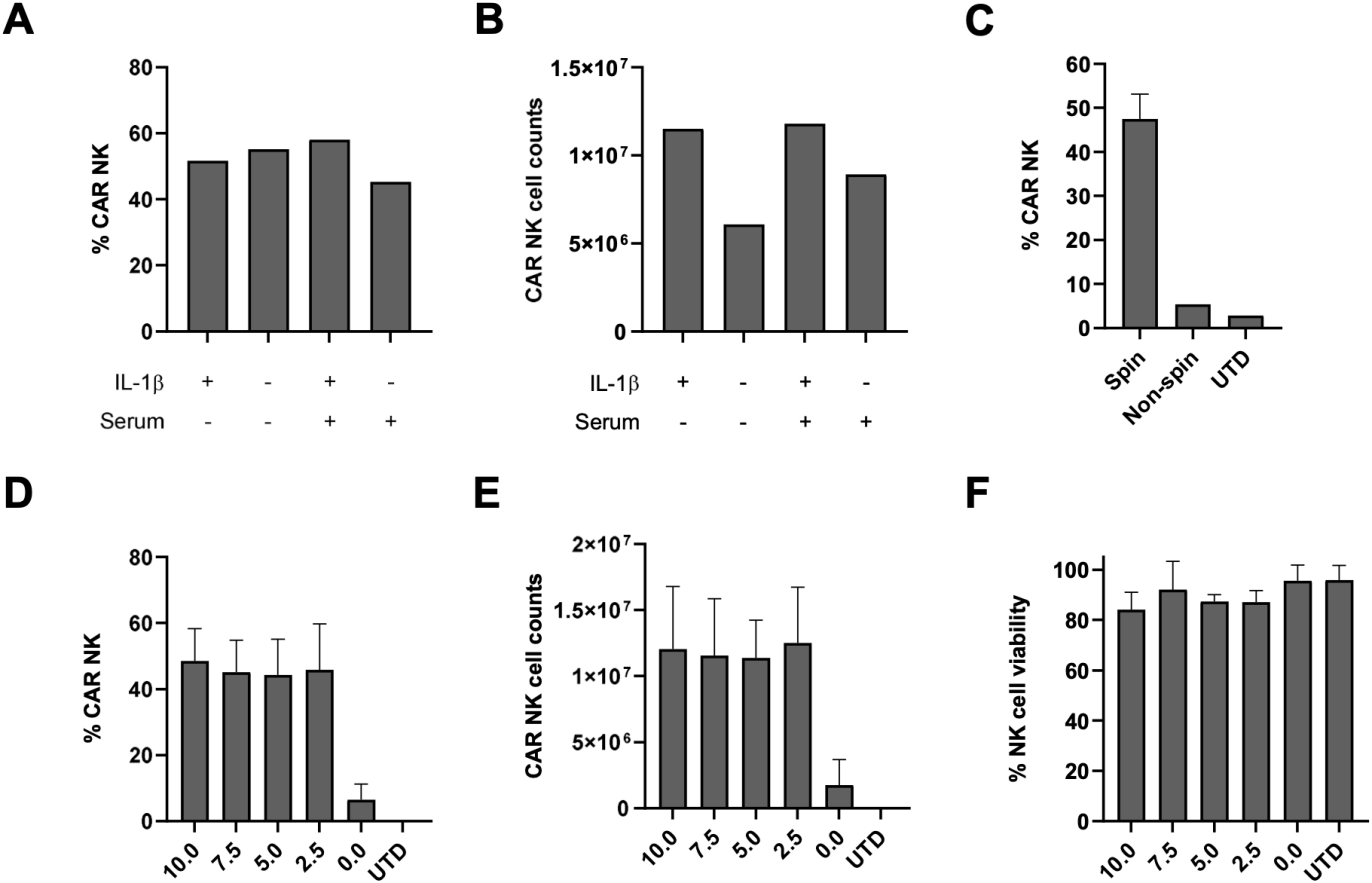
Assessment of factors affecting NK cell transduction. The impact of human AB serum and IL-1β on NK cell transduction efficiency **(A)** and expansion **(B)**. **(C)** The role of spinoculation in NK cell transduction. The effect of Vectofusin-1 concentrations on NK cell transduction efficiency **(D)**, cell counts **(E)** and viability **(F)**. In **(C–F)**, data are represented as mean ± SD from two donors in two independent experiments.

To enable automated NK cell transduction on the CliniMACS Prodigy platform, we first optimized the BaEV-LV-based transduction protocol for its compatibility. The impact of IL-1β and human AB serum on NK cell transduction efficiency was evaluated. Freshly isolated NK cells were activated for 2 days with IL-2 and IL-15, with or without IL-1β, in NK MACS Medium supplemented with 5% human AB serum. NK cells were then transduced with BDCA2 BaEV-LV in the presence or absence of human AB serum. Transduction efficiency was assessed 12 days post-transduction. Neither IL-1β nor human AB serum markedly influenced transduction efficiency (**Figure 1A**, **Supplementary Figure 1**). However, IL-1β activation enhanced CAR NK cell expansion compared to non-IL-1β activated controls (**Figure 1B**, **Supplementary Figure 1**). These results permit the omission of the serum-removal washing step prior to transduction. Subsequently, spinoculation was confirmed as essential to achieve efficient BaEV-LV-mediated NK cell transduction (**Figure 1C**). Lastly, the concentration of Vectofusin-1 was optimized during transduction. Although Vectofusin-1 substantially increased transduction efficiency, concentrations above 2.5 µg/mL did not yield further improvement (**Figure 1D**). Higher concentrations did not adversely affect CAR NK cell numbers and viability, indicating no cytotoxicity introduced by Vectofusin-1 (**Figures 1E, F**).

**Figure 2:**
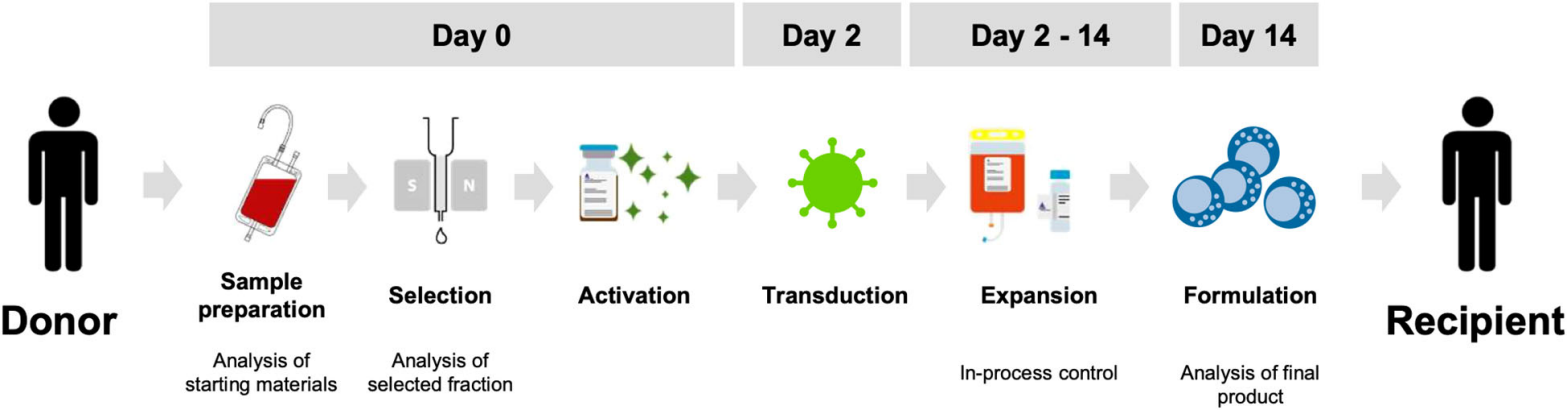
Workflow of the Natural Killer Cell Transduction (NKCT) process on the CliniMACS Prodigy platform. On day 0, following the preparation and analysis of leukapheresis, NK cells are isolated by magnetic selection through CD3 depletion using the CliniMACS Prodigy TS320 tubing set, followed by CD56 enrichment using the CliniMACS Prodigy TS520 tubing set. Isolated NK cells are then activated by cytokines. On day 2, NK cells are transduced and subsequently expanded for an additional 12 days. Routine in-process control (IPC) analyses are performed. Finally, on day 14, NK cells are harvested and ready for administration to recipients upon successful completion of quality control (QC) testing.

**Figure 3:**
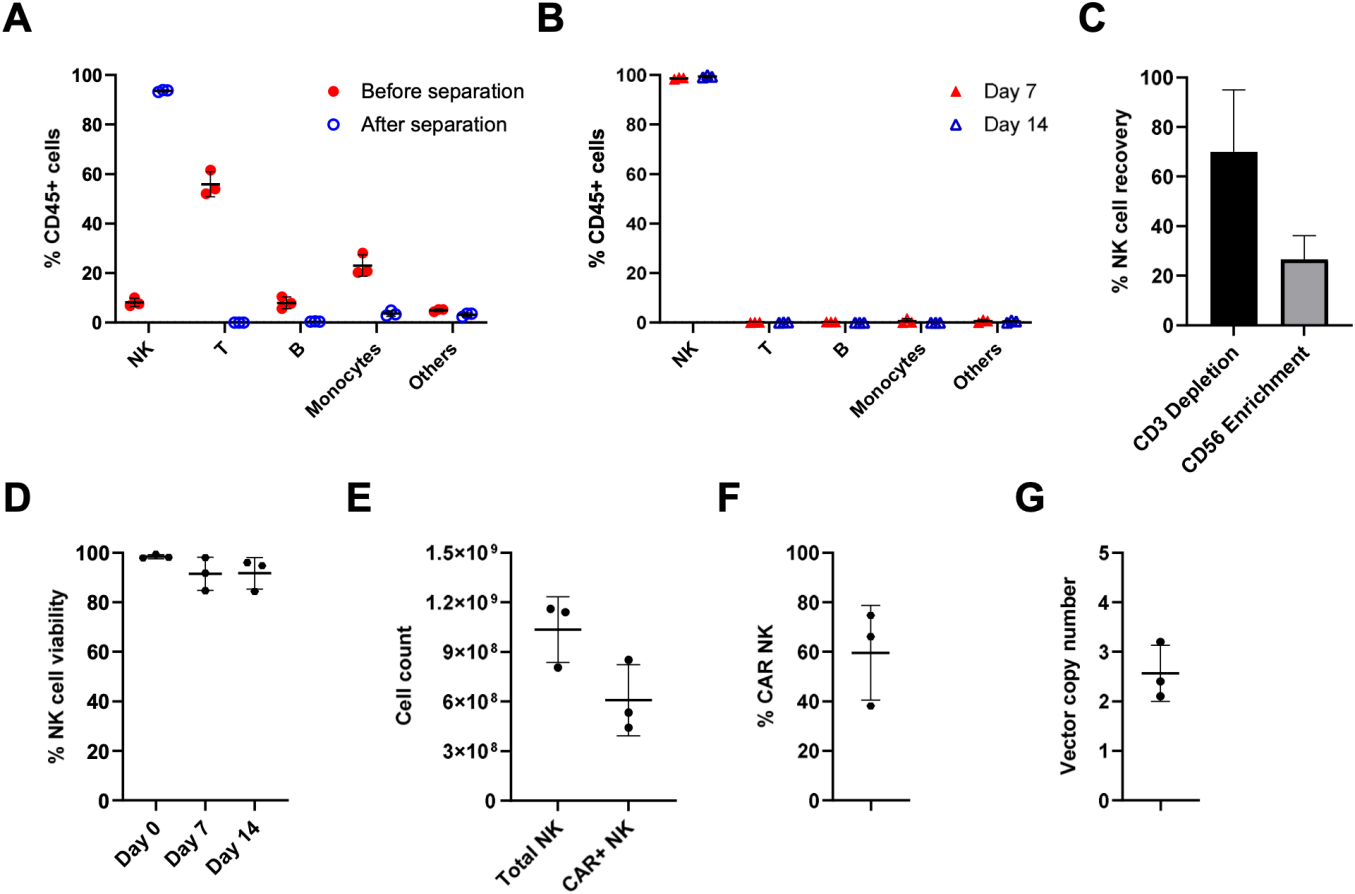
Automation of CAR NK cell manufacturing using the NKCT process. Three independent runs were performed using the NKCT process on the CliniMACS Prodigy platform. BDCA2 CAR NK cells were produced in these runs. The cellular composition was assessed by flow cytometry before and directly after separation **(A)**, as well as after 7 and 14 days of culture **(B)**. NK cell recovery during separation **(C)**, viability **(D)**, final cell count **(E)**, CAR expression **(F)**, and vector copy number **(G)** were also determined. Cells were gated as shown in **Supplementary Figures 6** and **7**. Data are represented as mean ± SD from three donors in three independent experiments.

The automated NKCT process was developed on the CliniMACS Prodigy platform to manufacture clinical-grade CAR NK cells (**Figure 2**). Before isolation, the concentration, volume, and proportions of CD3+ and CD56+ cells in a donor-derived leukapheresis product are assessed to set parameters for automated separation. NK cells are then selected via immunomagnetic depletion of CD3+ cells, followed by enrichment of CD56+ cells on day 0. After cytokine-based, feeder cell-free activation, NK cells are transduced by BaEV-LV on day 2 and expanded in NK MACS medium supplemented with IL-15 and IL-2 over 14 days. Following separation, NK cells became the predominant population, increasing from an average of 8.12% to 93.65%, with only residual T cells, B cells, monocytes, and other cells including granulocytes remaining (**Figure 3A**). NK cell purity further increased to 98.72% on day 7 and 99.45% on day 14 during the culture (**Figure 3B**). NK cell recovery rates after the CD3 depletion and the CD56 enrichment were 83.03% and 36.08%, respectively (**Figure 3C**). NK cells showed high viability of more than 91% on average throughout the process (**Figure 3D**). On day 14, an average of 1.04 x 10^9^ total NK cells were harvested at the end of the process (**Figure 3E**). BDCA2 BaEV-LV produced under cGMP conditions were used to generate CAR NK cells. The average NK cell transduction efficiency and vector copy number (VCN) of the CAR NK cell products were 59.64% and 2.57, respectively (**Figures 3F, G**).

### NKCT-manufactured BDCA2 CAR NK cells exhibit potent *in vitro* antitumor activity

**Figure 4:**
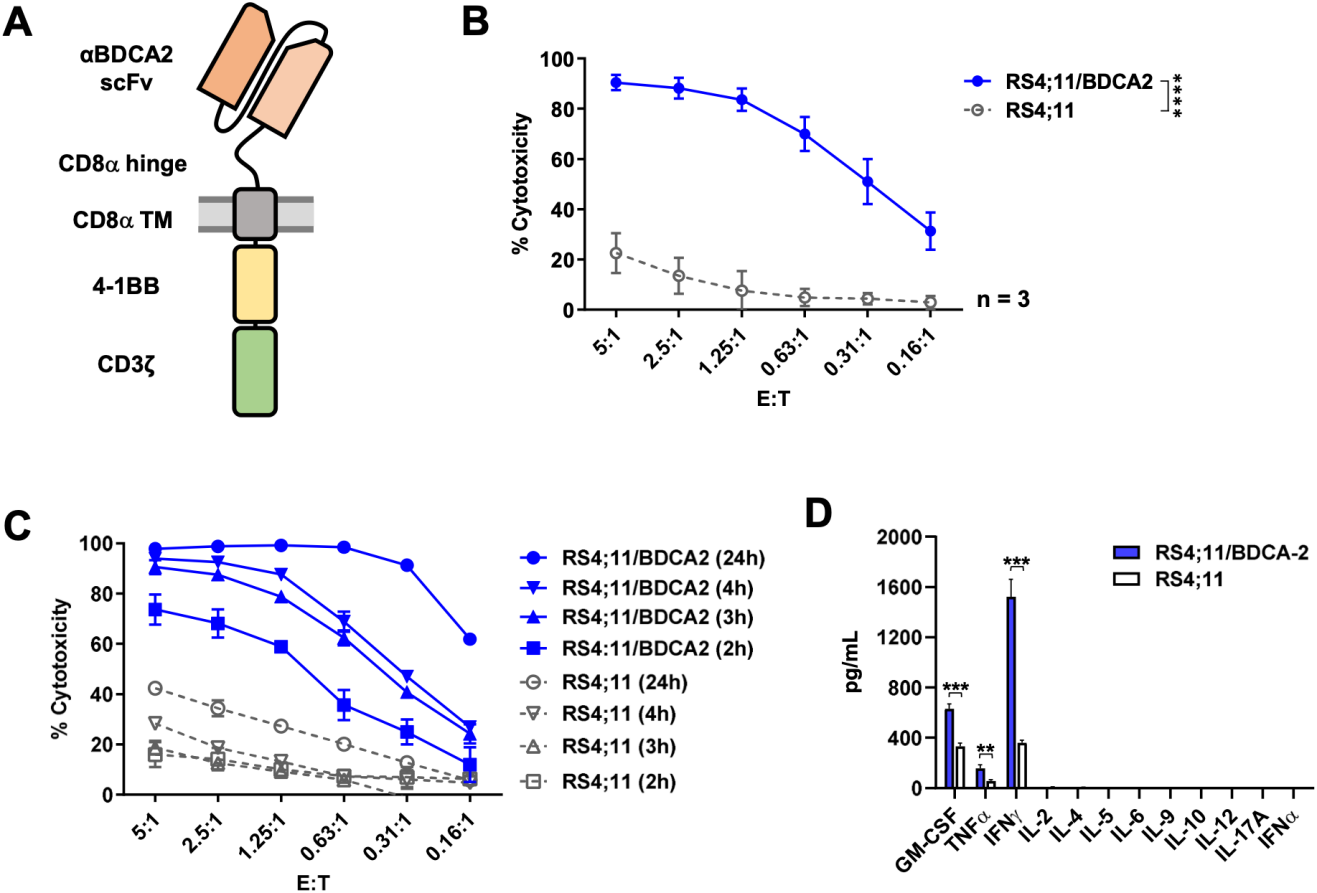
CliniMACS Prodigy-manufactured BDCA2 CAR NK cells exhibit potent antitumor activity *in vitro*. **(A)** Schematic representation of the BDCA2 CAR construct. **(B)** Cytotoxicity of BDCA2 CAR NK cells. BDCA2 CAR NK cells were co-incubated at the indicated E:T ratios with RS4;11/BDCA2 or RS4;11 tumor cells for 4 h Tumor cell lysis was quantified by flow cytometric analysis. Data are represented as mean ± SD from three donors in three independent experiments. Statistical significance was analyzed by two-way ANOVA. ****p < 0.0001. **(C)** Tumor lysis kinetics of BDCA2 CAR NK cells. BDCA2 CAR NK cells were co-incubated at the indicated E:T ratios with RS4;11/BDCA2 or RS4;11 tumor cells for 2, 3, 4 and 24 h. Tumor cell lysis was quantified by flow cytometric analysis. Data are represented as mean ± SD from three technical replicates. **(D)** Cytokine secretion of BDCA2 CAR NK cells. BDCA2 CAR NK cells were co-incubated at an E:T ratio of 1:1 with RS4;11/BDCA2 or RS4;11 tumor cells for 24 h Supernatants were collected and analyzed using a MACSPlex Cytokine 12 kit. Data are represented as mean ± SD from three technical replicates. Statistical significance was analyzed by two-tailed, unpaired Student’s t-test. ***p < 0.001; **p < 0.01.

To target BDCA2-expressing tumor cells, we designed and generated a second-generation BDCA2 CAR composed of a BDCA2-specific scFv, the CD8 hinge and transmembrane domains, and the intracellular domains of 4-1BB and CD3ζ (**Figure 4A**). The antitumor activity of BDCA2 CAR NK cells generated by the NKCT process on CliniMACS Prodigy was first evaluated *in vitro*. CAR NK cells were co-cultured with either RS4;11 or RS4;11/BDCA2 target tumor cells for 4 h at different E:T ratios. CAR NK cells displayed significantly higher cytotoxicity against BDCA2-expressing tumor cells, compared to BDCA2-negative tumor cells (**Figure 4B**). A prolonged coculture period further enhanced tumor cell lysis by CAR NK cells, eliminating 61% of tumor cells after 24 h at the lowest E:T ratio of 0.16:1 (**Figure 4C**). Furthermore, BDCA2 CAR NK cells secreted significantly higher levels of proinflammatory cytokines, including GM-CSF, IFNγ and TNFα, when cultured with RS4;11/BDCA2 cells compared to RS4;11 cells, demonstrating specific activation of CAR NK cells upon tumor target engagement (**Figure 4D**). No detectable secretion of IFNα, IL-2, IL-4, IL-5, IL-6, IL-9, IL-10, IL-12A or IL-17A was observed (**Figure 4D**).

### Supplementation with exogenous IL-2 potentiates the tumor-suppressing ability of BDCA2 CAR NK cells *in vivo*

The *in vivo* antitumor efficacy of CliniMACS Prodigy-manufactured BDCA2 CAR NK cells with the NKCT process was further evaluated in a xenograft mouse model. BDCA2 CAR NK cells and UTD NK cells derived from the same healthy donor were manufactured in parallel on two CliniMACS Prodigy devices using the NKCT process. After 14 days, a total of 4.09 x 10^9^ NK cells with a transduction efficiency of 34.69% were harvested for the CAR NK cell culture, while a total of 6.28 x 10^9^ UTD NK cells were produced (**Supplementary Figures 2A, B**). To assess the long-term effector potency of BDCA2 CAR NK cells used in the *in vivo* experiment, UTD NK cells or CAR NK cells were cultured with 3 x 10^4^ RS4;11/BDCA2 tumor cells at an initial E:T ratio of 1:2. NK cells were then rechallenged with 3 x 10^4^ fresh tumor cells every 24 h for 3 days. Over time, BDCA2 CAR NK cells exhibited significantly higher cytotoxicity against RS4;11/BDCA2 tumor cells compared to UTD NK cells (**Supplementary Figure 2C**).

**Figure 5:**
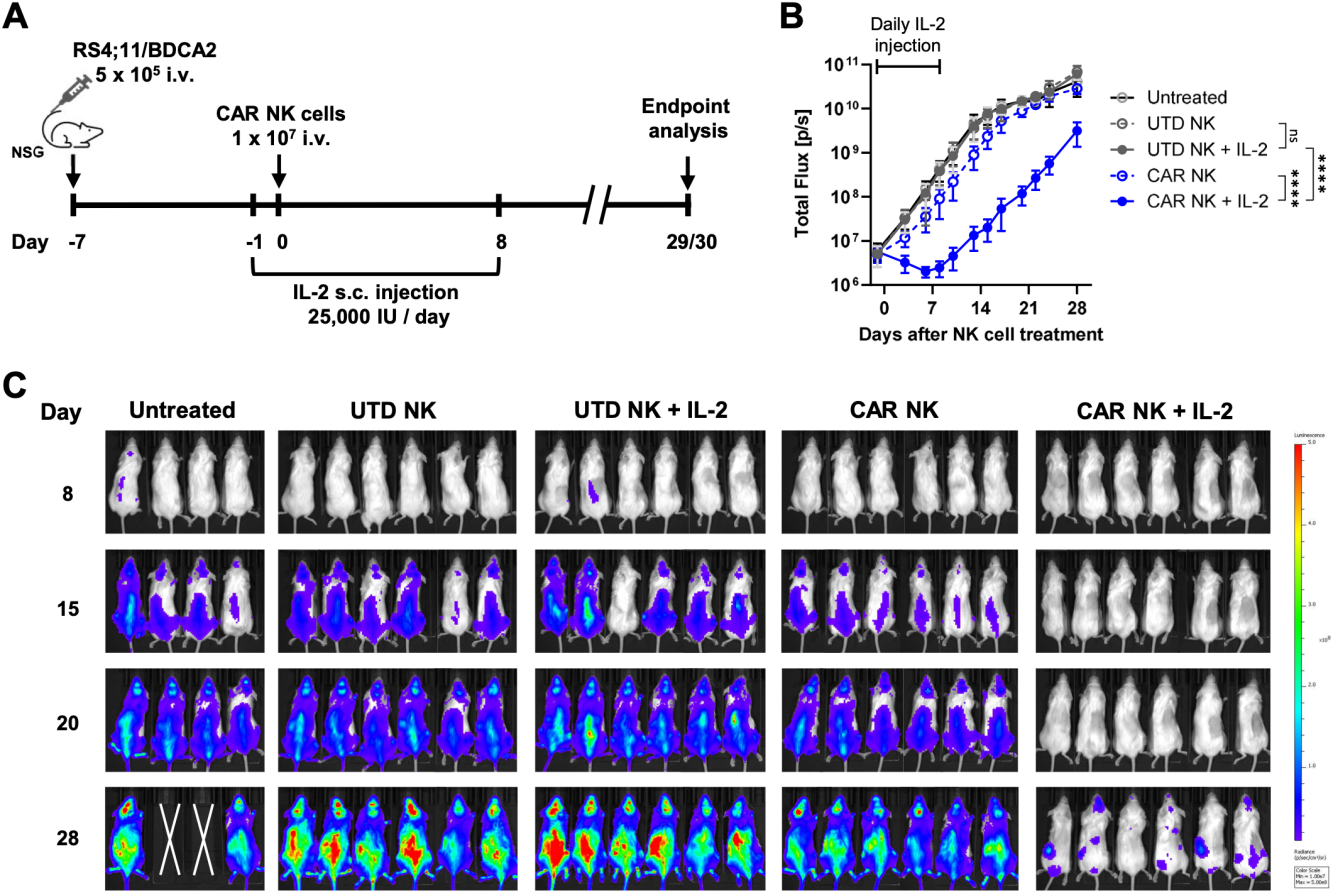
CliniMACS Prodigy-manufactured BDCA2 CAR NK cells strongly inhibit tumor growth *in vivo* when supplemented with exogenous IL-2. **(A)** On day -7, NSG mice received 5x10^5^ RS4;11/BDCA2 cells intravenously. On day 0, mice were intravenously injected with 1x10^7^ CAR NK cells or an equivalent number of UTD NK cells. Subcutaneous injections of IL-2 (25,000 U/day) were given to indicated mouse groups from day -1 to day 8. Tumor growth was monitored every two to three days using bioluminescence imaging (BLI). Total flux **(B)** and representative BLI images **(C)** were recorded. Data are represented as mean ± SD. Statistical significance was analyzed by two-way ANOVA. ****p < 0.0001; ns: *p* > 0.05.

NSG mice received 5x10^5^ RS4;11/BDCA2 tumor cells via the tail vein. Seven days later, mice were treated with either 1x10^7^ BDCA2 CAR NK cells or an equal number of UTD NK cells. To maintain NK cell persistence, daily subcutaneous injection of IL-2 was given to 2 cohorts of mice treated with UTD NK cells or CAR NK cells (**Figure 5A**). Prior to NK cell infusion, mice were randomized into five treatment groups, all of which exhibited comparable tumor burden (**Supplementary Figure 3**). In the untreated and UTD NK cell groups, tumor growth rapidly progressed regardless of IL-2 support. Infusion of CAR NK cells without IL-2 support resulted in only a slight delay in tumor progression (**Figures 5B, C**). On the contrary, CAR NK cells combined with IL-2 treatment led to significant tumor regression, although all mice eventually relapsed when IL-2 support was withdrawn (**Figures 5B, C**).

**Figure 6:**
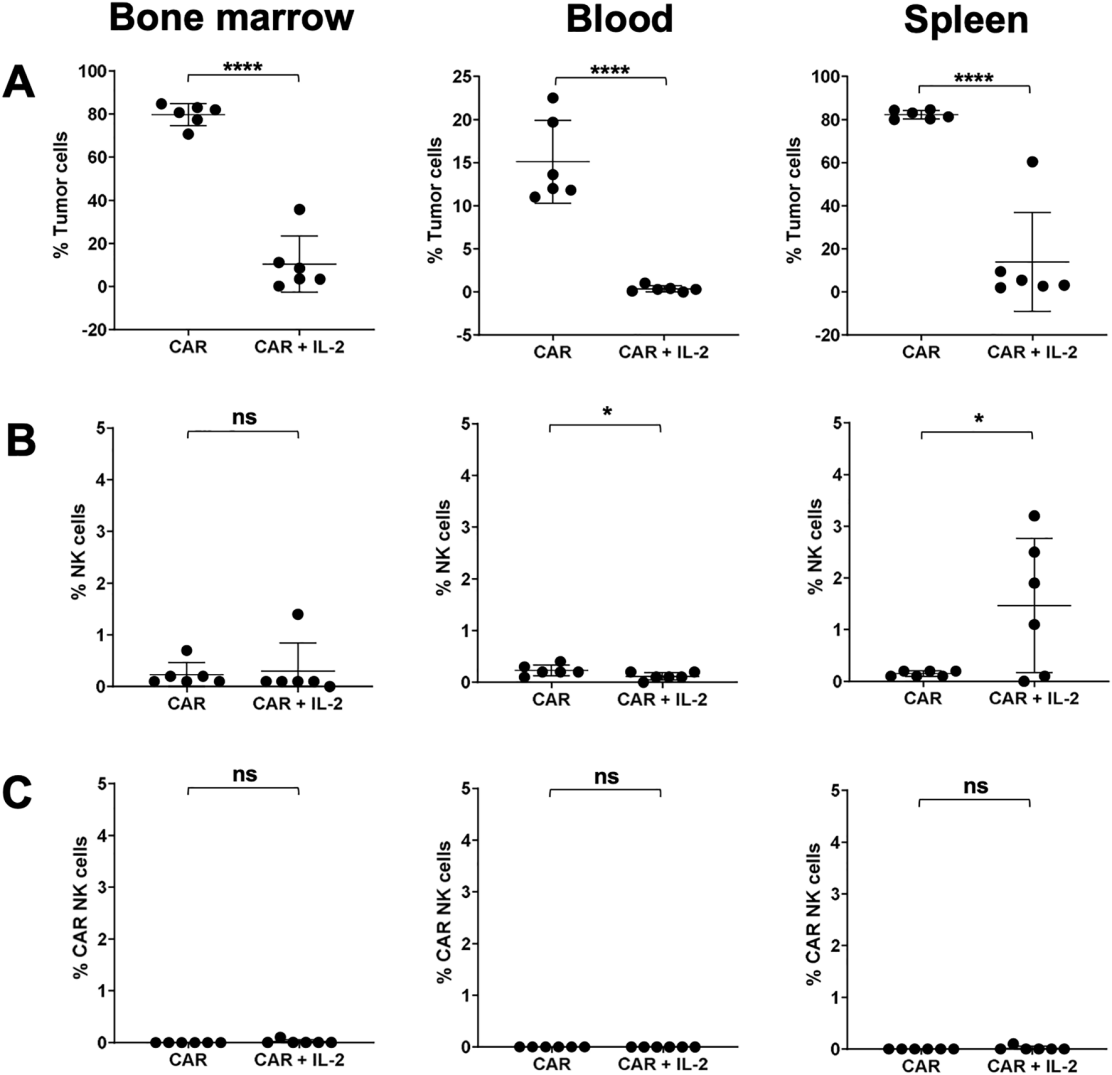
Flow cytometric analysis of the *in vivo* persistence of tumor cells and NK cells. At the conclusion of the experiment, the persistence of tumor cells **(A)**, NK cells **(B)**, and CAR NK cells **(C)** in the bone marrow, blood and spleen was determined by flow cytometry for mice treated with CAR NK cells with or without exogenous IL-2 support. Data are represented as mean ± SD. Statistical significance was analyzed by two-tailed, unpaired Student’s t-test. ****p < 0.0001; *p < 0.05; ns: *p* > 0.05.

Consistent with these findings, mice treated with CAR NK cells without IL-2 support had high tumor loads in the bone marrow (BM), blood, and spleen at the end of the study, while those receiving CAR NK cells with IL-2 support showed significantly reduced tumor burden (**Figure 6A**). No pronounced persistence of NK cells or CAR NK cells was observed in the BM, blood or spleen of mice treated with CAR NK cells without IL-2 support. However, a small population of human NK cells was found in the spleens of 4 out of 6 mice treated with CAR NK cells and IL-2 (**Figures 6B, C**). These persistent NK cells did not exhibit detectable CAR expression on their cell surfaces (**Figures 6B, C**). No BDCA2 tumor antigen loss were observed in mice treated with CAR NK cells (**Supplementary Figure 4**). No weight loss or other abnormalities were observed in mice during the experiment, indicating the interventions were well tolerated (**Supplementary Figure 5**).

## Discussion

Though rare, BPDCN is an aggressive hematological malignancy that is derived from plasmacytoid dendritic cell precursors. The prognosis for patients treated with conventional chemotherapy and radiotherapy is poor, with overall survival remaining dismal. The development of CD123-targeted therapies has shown promising clinical outcomes and led to the FDA approval of tagraxofusp, the first targeted therapy for BPDCN (17). Despite its benefits, this CD123-targeted regimen carries a substantial risk of capillary leak syndrome as a severe adverse event linked to patient mortality (18). This may be due to the expression of CD123 on endothelial cells (19). BDCA2 has been identified on the surface of plasmacytoid dendritic cells and represents a novel target for BPDCN treatment (20). Here, we developed novel CAR NK cells expressing a second generation BDCA2-specific CAR. Due to their low risk of causing graft-versus-host disease (GVHD), severe cytokine release syndrome (CRS), and neurotoxicity, CAR NK cells offer an excellent safety profile, making them ideal for advancing cellular therapies (3).

The traditional manufacturing of CAR NK cells is a complicated and labor-intensive procedure involving multiple manual steps, often conducted in open systems, particularly during the gene-engineering phase. A previous study by Oberschmidt et al. successfully established an automated process on the CliniMACS Prodigy system for producing clinical-grade NK cells, including cell separation and expansion; however, it did not support the genetic modification of NK cells (9). To simplify and standardize CAR NK cell manufacturing for clinical applications, we developed a cGMP-compliant, automated NKCT process within the closed CliniMACS Prodigy system that includes an integrated feature of NK cell lentiviral transduction. The cell separation using an automated set of commands (the NKCT process), that features CD3 depletion followed by CD56 enrichment using immunomagnetic nanoparticles, yielding a cell population composed primarily of NK cells, with only minimal contamination from other immune cells. Following cytokine-based *ex vivo* activation, purified NK cells were transduced with BaEV-LV using MACS GMP Vectofusin-1, a soluble enhancer designed to boost transduction efficiency. Vectofusin-1 enables the transduction of NK cells with BaEV-LV without the need for pre-coating culture plates or vessels with RetroNectin or fibronectin, which is typically required for retroviral vector transduction (10, 21). This automated viral transduction feature in the NKCT process led to a high transduction efficiency of NK cells. Of note, the average VCN was 2.57 across all runs, with no individual run surpassing the regulatory limit of a VCN of 5. After a two-week manufacturing procedure, the NKCT process yielded final CAR NK cell products that were highly viable and pure. This near complete removal of T lymphocytes in the final CAR NK cell product lowers the possibility of GVHD in recipients in allogeneic settings.

In addition to demonstrating the methodological effectiveness of our automated process, the antitumor activity of CliniMACS Prodigy-manufactured BDCA2 CAR NK cells was pronounced. High cytotoxicity and enhanced proinflammation cytokine release of BDCA2 CAR NK cells was shown in the *in vitro* assays against RS4;11/BDCA2 tumor cells that are otherwise largely resistant to NK cells (**Figure 4B**). Although BDCA2 CAR NK cells were able to slightly suppress tumor cell growth in mice compared to UTD NK cells, their *in vivo* antitumor activity was demonstrated only when exogenous IL-2 support was present. Tumor regression was observed in mice treated with BDCA2 CAR NK cells during the period of exogeneous IL-2 support; however, the tumor burden increased rapidly once the cytokine treatment was stopped. This is likely due to the *in vivo* persistence of NK cells being entirely dependent on exogenous cytokines, since NK cells, unlike T cells, do not secrete endogenous cytokines (notable IL-2) to support their survival and proliferation. Although this may restrict the persistence of NK cells after infusion in a clinical setting, limited persistence may also offer a safety benefit for CAR NK cells, particularly when the target is also present on healthy tissues. Various approaches to improving *in vivo* NK cell persistence have been explored in preclinical and clinical studies, such as engineering CAR NK cells to express cytokines in an autocrine manner or administering CAR NK cells along with IL-2 or IL-15 superagonists (5, 22–25).

In conclusion, the cGMP-compliant NKCT process on the closed CliniMACS Prodigy system provides a ready-to-use, automated platform, facilitating both centralized and decentralized manufacturing of clinical-grade CAR NK cells. It is highly adaptable for generating CAR NK cells for other cancer treatments (26). Combined with GMP-grade BaEV-LV, the NKCT process will enable the smooth and efficient clinical translation of gene-engineered NK cell therapeutics. In addition, CliniMACS Prodigy-manufactured BDCA2 CAR NK cells demonstrate potent antitumor activity against BDCA2-expressing tumor cells, suggesting a promising therapeutic approach for BPDCN.

## Data Availability

The original contributions presented in the study are included in the article/**Supplementary Material**. Further inquiries can be directed to the corresponding author.

## Supplementary material

Supplementary Figure 1 The impact IL-1β on NK cell transduction efficiency and expansion. Data are represented as mean ± SD from three donors in three independent experiments.

Supplementary Figure 2 Characterization of CliniMACS Prodigy-manufactured CAR NK cells used in the *in vivo* efficacy study. **(A)** UTD and BDCA2 CAR NK cells have been manufactured using the NKCT process on the CliniMACS Prodigy system and cell counts were recorded on days 7, 9 and 14. **(B)** CAR expression on day 14 was measured by flow cytometry. **(C)** The sustained cytotoxic activity of BDCA2 CAR NK cells against RS4;11/BDCA2 tumor cells was evaluated using an *in vitro* tumor rechallenge assay. UTD or CAR NK cells were co-incubated with tumor cells at an initial E:T ratio of 1:2 followed by daily rechallenge with fresh tumor cells for 3 days. Tumor cell lysis was quantified by flow cytometry. Data are represented as mean ± SD from three technical replicates. Statistical significance was analyzed by two-tailed, unpaired Student’s t-test. *****p* < 0.0001; **p* < 0.05.

Supplementary Figure 3 Randomization of animal groups for the *in vivo* experiment. One day prior to NK cell treatment, tumor cell engraftment in mice was confirmed using bioluminescent imaging. Subsequently, mice were randomized into five treatment groups with comparable tumor burden.

Supplementary Figure 4 Flow cytometric analysis of BDCA2 expression on tumor cells isolated from the bone marrow of mice that received different NK cell treatments. Each dot represents an individual mouse from respective groups. Data are represented as mean ± SD.

Supplementary Figure 5 Individual mouse weights were monitored throughout the *in vivo* experiment.

Supplementary Figure 6 Gating strategy used for analyzing cellular composition.

Supplementary Figure 7 Gating strategy used for analyzing transduction efficiency of CAR NK cells.
