# Supplementary figures and images for: Automated manufacturing of clinical-grade BDCA2 CAR NK cells in a closed system for the treatment of blastic plasmacytoid dendritic cell neoplasm
Source: Front Immunol. 2026 Feb 27;17:1761397. doi: 10.3389/fimmu.2026.1761397 (PMC12982444; doi:10.3389/fimmu.2026.1761397)

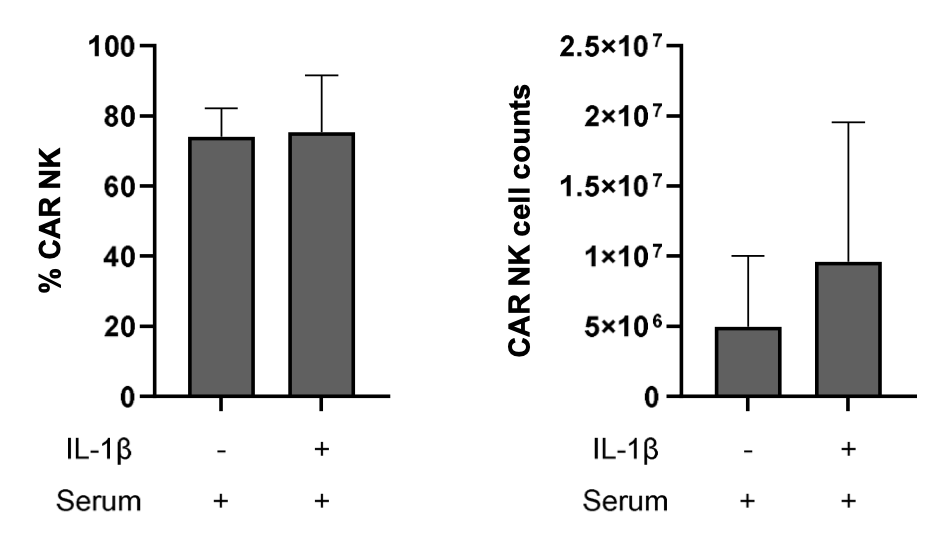

Supplement: Supplementary Figure 1 — The impact IL-1β on NK cell transduction efficiency and expansion. Data are represented as mean ± SD from three donors in three independent experiments. [file Image1.tiff]

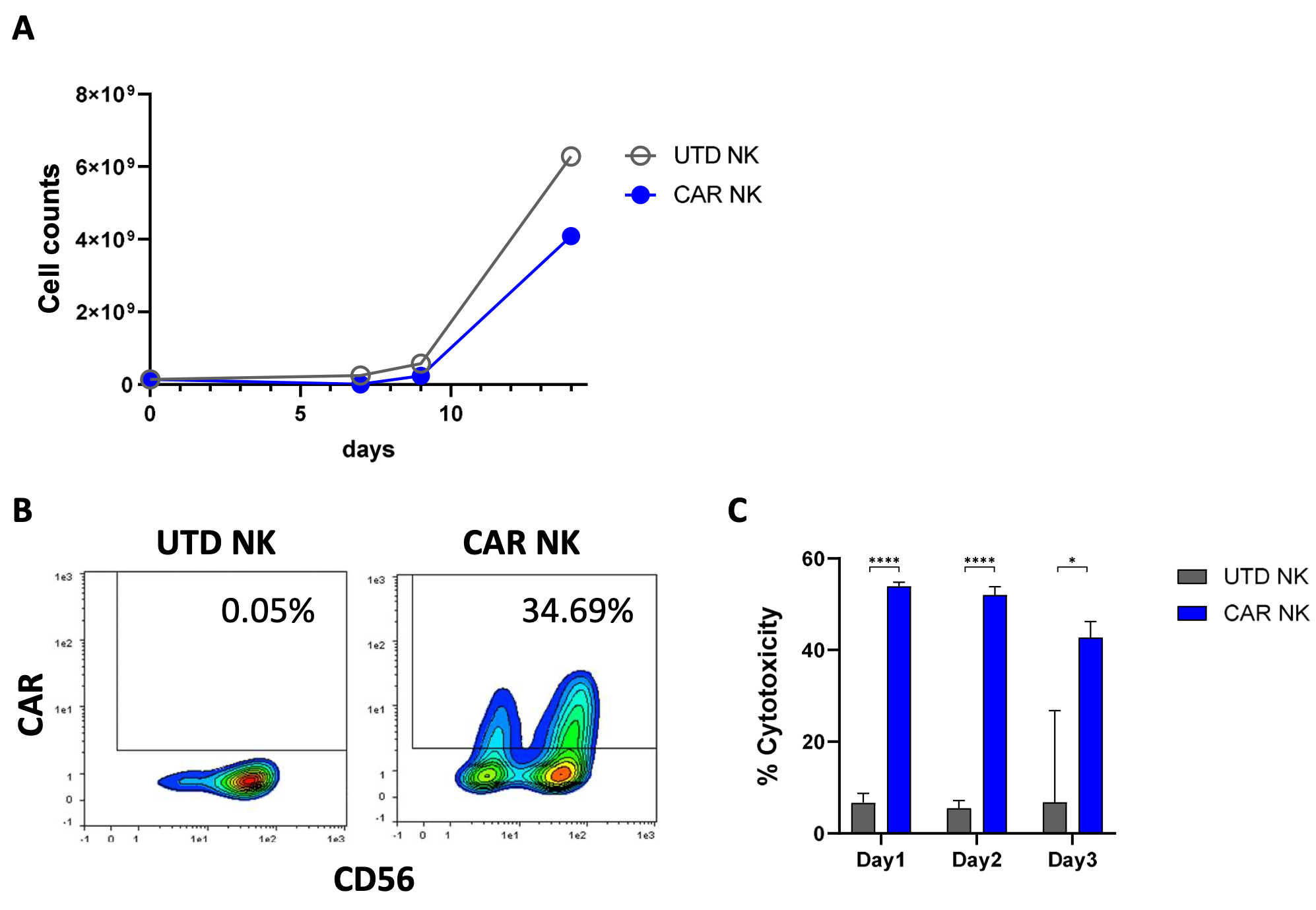

Supplement: Supplementary Figure 2 — Characterization of CliniMACS Prodigy-manufactured CAR NK cells used in the in vivo efficacy study. (A) UTD and BDCA2 CAR NK cells have been manufactured using the NKCT process on the CliniMACS Prodigy system and cell counts were recorded on days 7, 9 and 14. (B) CAR expression on day 14 was measured by flow cytometry. (C) The sustained cytotoxic activity of BDCA2 CAR NK cells against RS4;11/BDCA2 tumor cells was evaluated using an in vitro tumor rechallenge assay. UTD or CAR NK cells were co-incubated with tumor cells at an initial E:T ratio of 1:2 followed by daily rechallenge with fresh tumor cells for 3 days. Tumor cell lysis was quantified by flow cytometry. Data are represented as mean ± SD from three technical replicates. Statistical significance was analyzed by two-tailed, unpaired Student’s t-test. ****p < 0.0001; *p < 0.05. [file Image2.tiff]

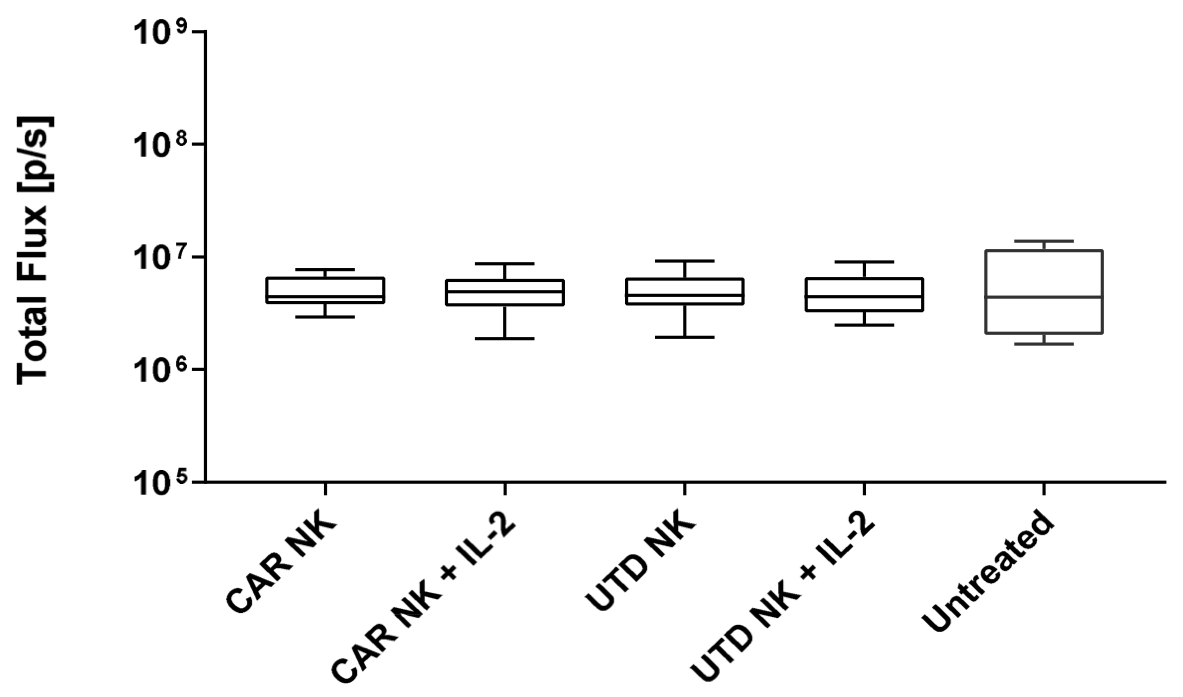

Supplement: Supplementary Figure 3 — Randomization of animal groups for the in vivo experiment. One day prior to NK cell treatment, tumor cell engraftment in mice was confirmed using bioluminescent imaging. Subsequently, mice were randomized into five treatment groups with comparable tumor burden. [file Image3.tiff]

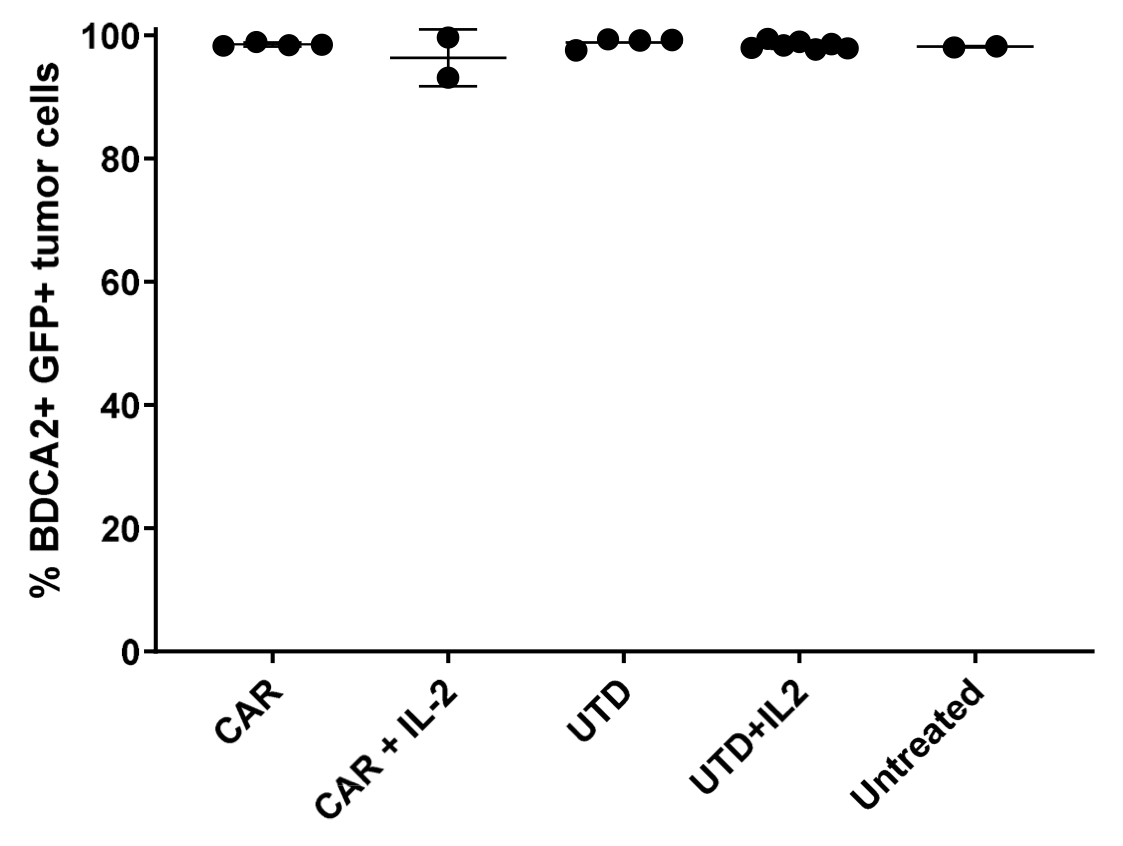

Supplement: Supplementary Figure 4 — Flow cytometric analysis of BDCA2 expression on tumor cells isolated from the bone marrow of mice that received different NK cell treatments. Each dot represents an individual mouse from respective groups. Data are represented as mean ± SD. [file Image4.tiff]

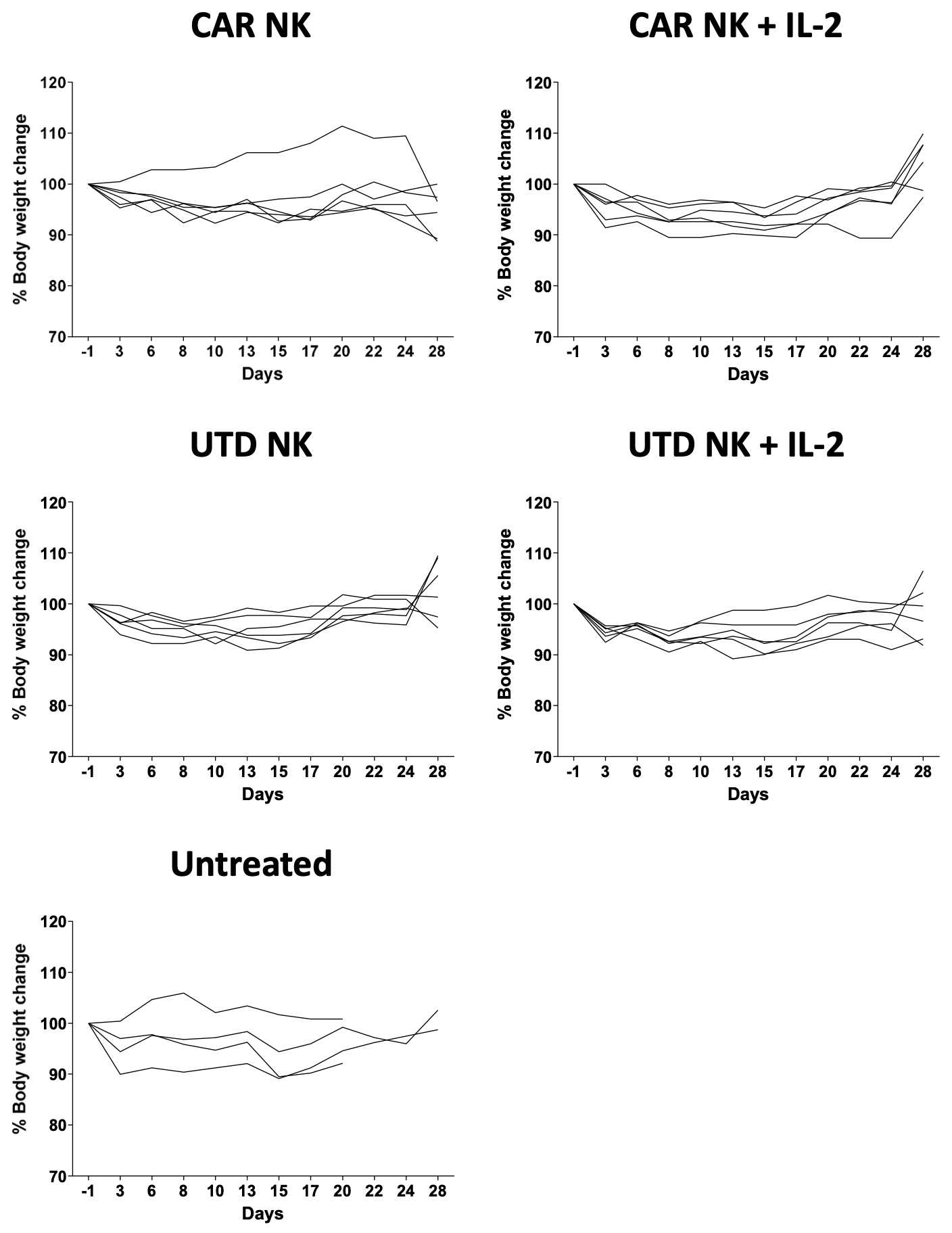

Supplement: Supplementary Figure 5 — Individual mouse weights were monitored throughout the in vivo experiment. [file Image5.tiff]

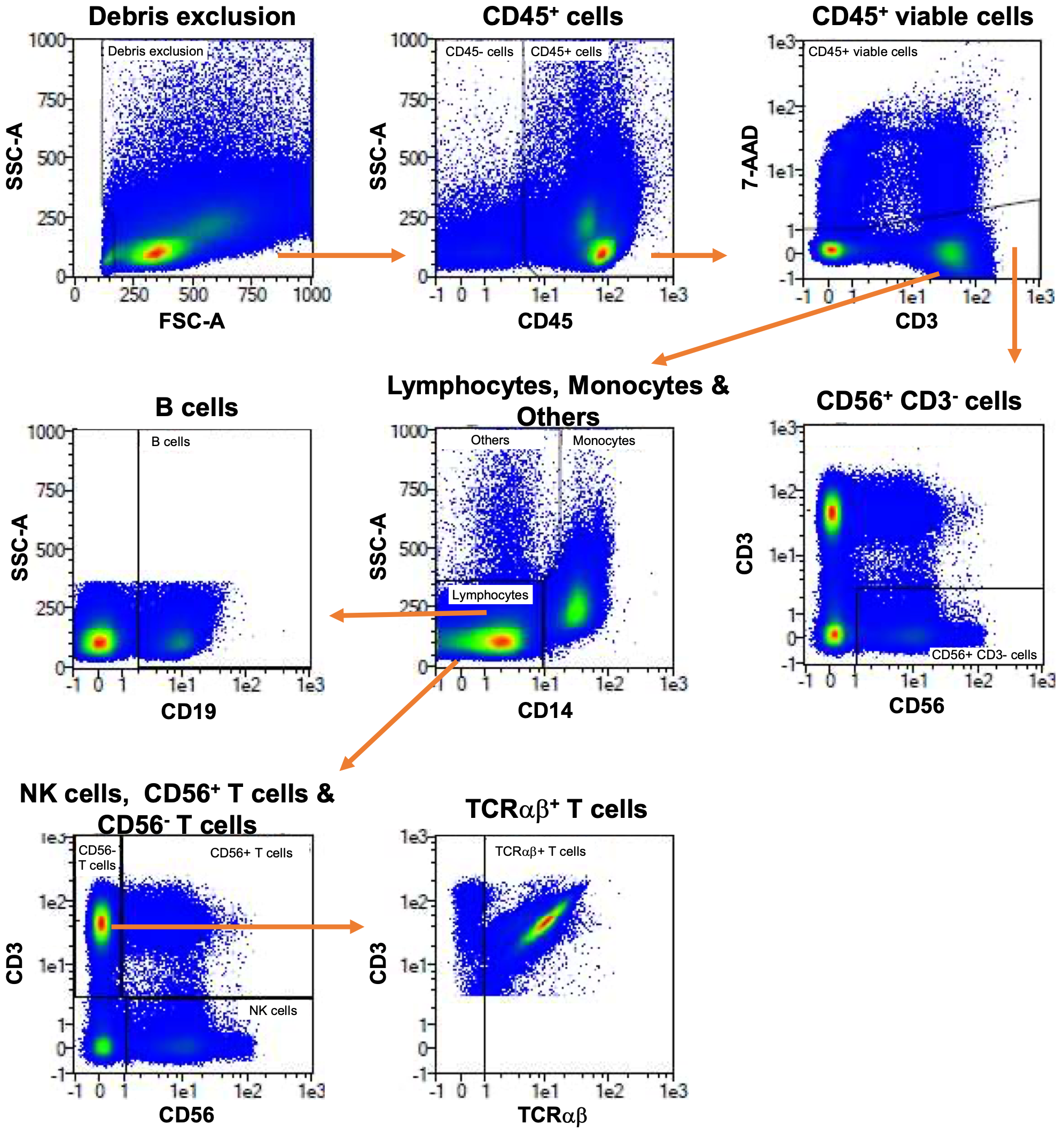

Supplement: Supplementary Figure 6 — Gating strategy used for analyzing cellular composition. [file Image6.tiff]

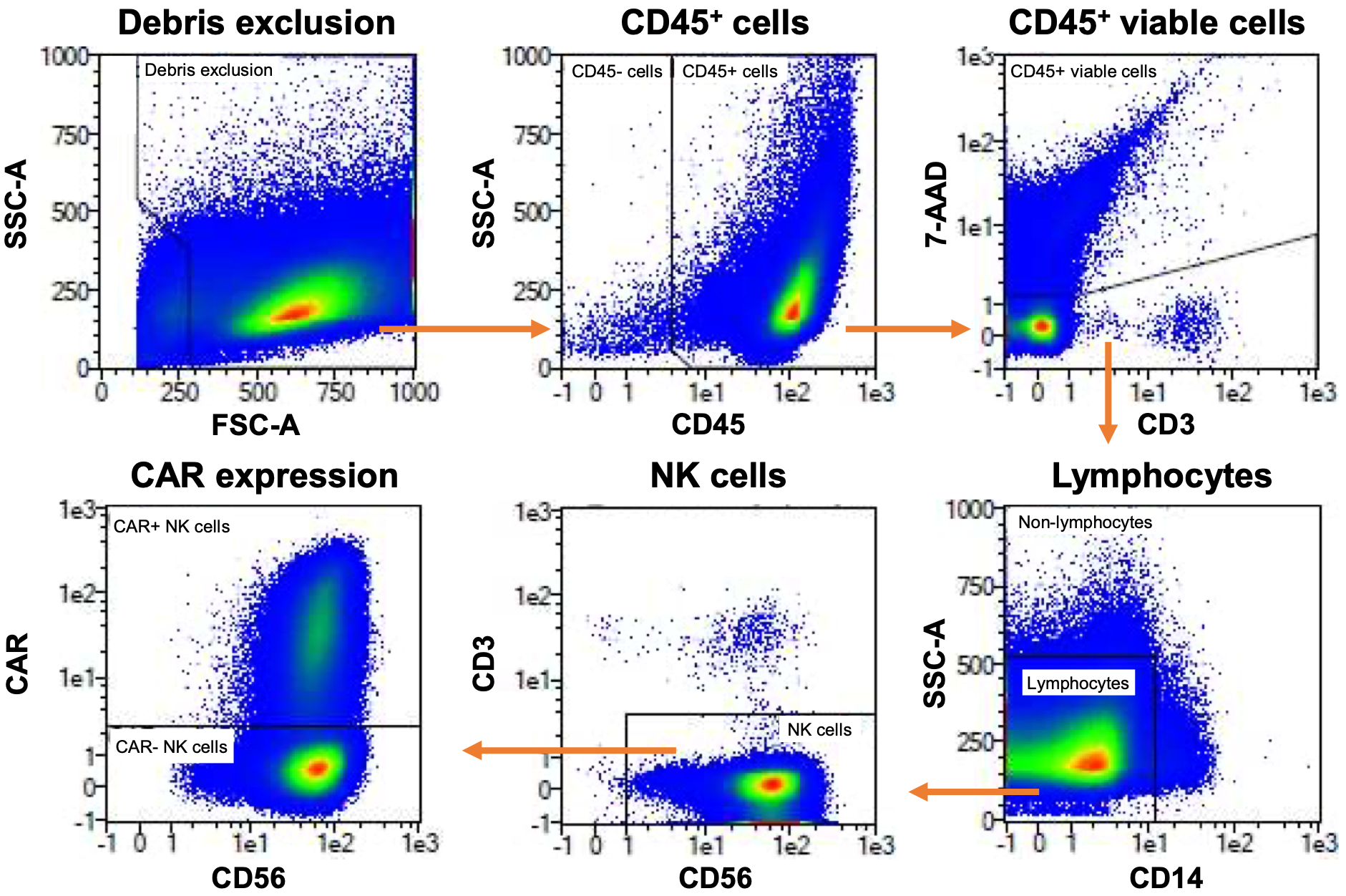

Supplement: Supplementary Figure 7 — Gating strategy used for analyzing transduction efficiency of CAR NK cells. [file Image7.tiff]
